# 'The difference in determinants of *Chlamydia trachomatis *and *Mycoplasma genitalium *in a sample of young Australian women.'

**DOI:** 10.1186/1471-2334-11-35

**Published:** 2011-02-01

**Authors:** Jennifer Walker, Christopher K Fairley, Catriona S Bradshaw, Sepehr N Tabrizi, Marcus Y Chen, Jimmy Twin, Nicole Taylor, Basil Donovan, John K Kaldor, Kathleen McNamee, Eve Urban, Sandra Walker, Marian Currie, Hudson Birden, Francis Bowden, Jane Gunn, Marie Pirotta, Lyle Gurrin, Veerakathy Harindra, Suzanne Garland, Jane S Hocking

**Affiliations:** 1 Centre for Women's Health, Gender and Society, School of Population Health, University of Melbourne, Victoria 3010, Australia; 2 Sexual Health Unit, School of Population Health, University of Melbourne, Victoria 3010, Australia; 3 Melbourne Sexual Health Centre, Alfred Health, Melbourne Victoria 3010, Australia; 4 Department of Epidemiology and Preventive Medicine, Monash University, Melbourne Victoria, Australia; 5 The Royal Women's Hospital, Parkville, Victoria, Australia; 6 National Centre in HIV Epidemiology and Clinical Research, UNSW, Sydney, Australia; 7 Family Planning Victoria, Melbourne, Australia; 8 Monash Medical Centre. Department of Obstetrics and Gynaecology, Clayton, Victoria, Australia; 9 Australian National University, Canberra, Australia; 10 North Coast Medical Education Collaboration, Sydney School of Public Health, University of Sydney, Lismore, NSW, Australia; 11 Primary Care Research Unit, Department of General Practice, University of Melbourne, Victoria 3010, Australia; 12 Centre for Molecular, Environmental, Genetic and Analytic Epidemiology, School of Population Health, University of Melbourne, Victoria 3010, Australia; 13 St Mary's Hospital, Portsmouth, UK; 14 Department of Obstetrics and Gynaecology, University of Melbourne, Victoria 3010, Australia

## Abstract

**Background:**

Differences in the determinants of *Chlamydia trachomatis *('chlamydia') and *Mycoplasma genitalium *(MG) genital infection in women are not well understood.

**Methods:**

A cohort study of 16 to 25 year old Australian women recruited from primary health care clinics, aimed to determine chlamydia and MG prevalence and incidence. Vaginal swabs collected at recruitment were used to measure chlamydia and MG prevalence, organism-load and chlamydia-serovar a cross-sectional analysis undertaken on the baseline results is presented here.

**Results:**

Of 1116 participants, chlamydia prevalence was 4.9% (95% CI: 2.9, 7.0) (n = 55) and MG prevalence was 2.4% (95% CI: 1.5, 3.3) (n = 27). Differences in the determinants were found - chlamydia not MG, was associated with younger age [AOR:0.9 (95% CI: 0.8, 1.0)] and recent antibiotic use [AOR:0.4 (95% CI: 0.2, 1.0)], and MG not chlamydia was associated with symptoms [AOR:2.1 (95% CI: 1.1, 4.0)]. Having two or more partners in last 12 months was more strongly associated with chlamydia [AOR:6.4 (95% CI: 3.6, 11.3)] than MG [AOR:2.2 (95% CI: 1.0, 4.6)] but unprotected sex with three or more partners was less strongly associated with chlamydia [AOR:3.1 (95%CI: 1.0, 9.5)] than MG [AOR:16.6 (95%CI: 2.0, 138.0)]. Median organism load for MG was 100 times lower (5.7 × 10^4^/swab) than chlamydia (5.6 × 10^6^/swab) (p < 0.01) and not associated with age or symptoms for chlamydia or MG.

**Conclusions:**

These results demonstrate significant chlamydia and MG prevalence in Australian women, and suggest that the differences in strengths of association between numbers of sexual partners and unprotected sex and chlamydia and MG might be due to differences in the transmission dynamics between these infections.

## Background

Genital *Chlamydia trachomatis *('chlamydia') infection is a significant public health problem among young Australian women, with notification rates increasing from 74 per 100 000 people per year in 1997, to 287 per 100 000 people per year in 2009 [1]. Prevalence estimates among young Australian women range from 3% to 5% in community-based samples [2,3], but these estimates are based on small sample sizes with limited precision. In light of Australia's future national chlamydia testing pilot program [4], there is an urgent need for reliable chlamydia prevalence estimates that can be used to both inform the design of the pilot and monitor its performance.

*Mycoplasma genitalium *(MG) is another important sexually transmitted pathogen that is associated with urethritis [5], cervicitis, endometritis [6], pelvic inflammatory disease (PID), tubal factor infertility [7], and an increased risk of HIV transmission [8]. Recent studies report varying prevalence estimates for MG in women; 0.8% (95% confidence interval [CI]:0.4, 1.6) among 18-27 year old sexually-active women in the US [9]; 2.3% (95% CI:1.3, 3.2) in 21-23 year old women Denmark [10]; and 3.4% (95% CI:2.7, 4.3) in sexually-active students in the UK [11]. However, differences in the type of specimens (urine or swabs) and if applicable, how the specimens were stored may contribute to these differences in prevalence [12]. Overall, MG prevalence has been found to be consistently lower than chlamydia [9-11]. In Australia, MG testing is not widely available and there are no population data. Understanding the burden of disease that might be attributable to MG in young Australian women is necessary to inform clinical practice and policy.

Our paper compares the epidemiological characteristics of chlamydia and MG in a community-based sample of sexually-active young women in order to gain insights into the epidemiology and transmission dynamics of these two infections within the same population. We examine prevalence estimates and explore clinical and behavioral factors associated with each infection. We also present chlamydia and MG organism loads and identified chlamydia serotypes.

## Methods

### Recruitment

The data presented in this paper were collected as part of a longitudinal study of young women - the Chlamydia Incidence and Re-infection Rates Study (CIRIS). The primary aim of CIRIS was to measure chlamydia incidence over 12 months; a secondary aim was to measure MG incidence. Women were recruited from 29 primary health clinics (including general practice, sexual health and family planning clinics) in three states in Australia between May 2007 and August 2008. Women were eligible for inclusion if they were aged 16 to 25 years, had ever had vaginal sex, were not pregnant at recruitment, and were able to be contacted by post within Australia during the 12 month study. A dedicated research assistant recruited consecutive women attending the clinic during a six week period. Informed consent was obtained for each participant prior to their recruitment into the study. Further methodological details are published elsewhere [13]. The prevalence estimates and other data presented here are based on testing and data collection at the time of recruitment (baseline).

### Testing

At baseline, each participant provided two self-collected vaginal swabs. One swab was tested for chlamydia by the clinic's preferred pathology testing laboratory using nucleic acid amplification techniques (NAAT). The second swab, a flocked swab http://www.microrheologics.com, was forwarded to Royal Women's Hospital (RWH), Melbourne, Victoria for MG testing and further studies including organism load quantification and chlamydia serovar determination if diagnosed chlamydia positive.

MG testing was conducted by rotating the swab in 400 μl of PBS and 200 μl was extracted using the automated MagNA Pure LC (Roche Molecular Biochemical, Mannheim, Germany) with the DNA Isolation Kit 1 protocol. Detection of MG was performed using the extracted DNA amplified by real-time PCR targeting a 517 bp region of the 16 S rRNA gene [14] and human beta-globin was used as a measure of sample adequacy as an internal control to detect the presence of possible inhibitors [15]. Any remaining specimen was stored at -80°C. All the participants were tested for MG at baseline, six months and 12 months throughout the study period. The MG test results were validated by retesting all MG positive samples and retesting a random sample of 836 stored study samples from all the swabs collected during the study period, at the conclusion of the study using a real-time PCR assay that was directed at the adhesion protein gene (MgPa) [16,17]. This sample size was selected in order to obtain a precision of 4% around a sensitivity of 97%. We found a kappa value of 0.97 (95% CI: 0.94 to 1.01), (sensitivity 95.0% and specificity 99.6%) between the two assays [18]. Women were given a positive MG diagnosis if they tested positive using either assay; this accounted for any possible DNA degradation of the sample during the storage period, and also, in the absence of a clear gold standard for the diagnosis of MG, it was clinically important to treat all women who were MG positive by either assay.

### Organism load and serovars

Quantification of chlamydia load was determined by a quantitative PCR (qPCR) system targeting the *omp1 *gene using published methodology [19]. The chlamydial load in each tested sample was quantified by comparing the crossing-threshold of each sample to the crossing-threshold of a standard curve constructed by amplifying different known copy numbers of the *omp1 *gene. This method also determined whether any mixed infections were present, and identified the chlamydia serovar(s) of each infection through a series of qPCR assays using serovar-specific probes. Confirmation of each chlamydia serovar, and detection of genotypic variants were determined by DNA sequencing across all four variable domains of the *omp1 *gene that encodes for the antigenic major outer membrane protein as previously described [20].

The MG concentration of each sample was quantified using a qPCR (TaqMan^® ^MGB Probe) assay targeting the *MgPa *gene [21]. Quantification was carried out using a LightCycler 480 Real-Time PCR System (Roche Diagnostics, Mannheim, Germany) by comparing the quantification cycle of each sample to the quantification cycle of a standard curve constructed by amplifying different known copy numbers of target gene. Organism loads were presented as copies per swab.

### Management of participants

Women who tested positive for chlamydia at baseline were managed by the clinic from where they were recruited. The treating clinicians were provided with up-to-date chlamydia treatment guidelines (1 g of azithromycin for uncomplicated chlamydia infection) and partner notification material [22]. Subsequent follow-up surveys determined if treatment had been taken and if partner(s) had been treated. All women who tested positive were asked to re-test for chlamydia three months after treatment.

All women who tested positive for MG were managed by the research team and a sexual health physician. Clinical symptoms and partner notification were discussed and support material and partner notification letters were provided. Treatment with 1 g of azithromycin [23] was provided if there were no symptoms to suggest PID. Women were sent a second vaginal swab for a test-of-cure one month following treatment. If the test-of-cure was positive and no risk of re-infection was identified via telephone consultation, the patient was treated with 400 mg moxifloxacin daily for 10 days [24], otherwise a repeat 1 g dose of azithromycin was prescribed [24] and another test-of-cure was done a month later.

### Data collection

Women were asked to complete a self-administered questionnaire at recruitment. This collected demographic, sexual behaviour data (including number of sex partners), and recent antibiotic and contraceptive use. It also included questions about the presence of any genital symptoms during the month prior to recruitment, including abnormal vaginal discharge and pelvic pain.

### Statistical methods

Power calculations assuming a design effect of 2 suggested that a sample size of 1,000 would be sufficient to generate standard error of 0.8% and 0.6% for prevalence estimates of 5% and 2% respectively.

Data were analysed using STATA version 10.2 [25]. All analyses were adjusted for clustering at the clinic level and for type of clinic (general practice versus sexual health/family planning clinic). Prevalence estimates and 95%confidence intervals (95% CIs) were calculated and odds ratios (OR) and robust standard errors were calculated to explore associations with chlamydia and MG. For the analysis of the associations with chlamydia or MG and symptoms, only women who had tested positive for one infection were included and women with a co-infection were excluded. Associations with organism load for both chlamydia and MG were explored using linear regression and organism load was logarithm transformed because of the skewed distribution of the raw data.

Ethics approval to conduct this study was obtained from ten Human Research Ethics Committees throughout Australia.

## Results

### Characteristics of sample

Overall, 66% of consecutive, eligible women agreed to participate in the study (n = 1116) with two-thirds recruited from general practice clinics (20 out of 29 clinics). The participants had a median age of 21 years, and when compared with the most recent Australian census data for women in the same age group, the study participants were more likely to be Australian-born (89% versus 79%, *p *< 0.01) [26] and more well-educated (tertiary degree 44% versus 21% *p *< 0.01) [26]. Compared with women of the same age in the 'Australian Study of Health and Relationships' (a nationally representative sexual behaviour survey), women in our study were more likely to report having had three or more sexual partners in the last 12 months (33% versus 9.5%, *p *< 0.01) [27]. There were no differences for all other reported demographics according to the Australian Bureau of Statistics census data [26].

### Prevalence estimates and associations

A total of 55 women tested positive for chlamydia [prevalence: 4.9% (95% CI:2.9, 7.0)] and 27 tested positive for MG [prevalence: 2.4% (95% CI:1.5, 3.3)]. Two women were co-infected with both chlamydia and MG [0.2% (95% CI:0.0, 0.4)]. Prevalence estimates were higher among women recruited from sexual health clinics than from general practice clinics for both chlamydia [7.9% (95% CI:4.1, 11.8) compared with 3.4% (95% CI:1.5, 5.3) (*p *= 0.01)], and MG [4.0% (95% CI:2.7, 5.3) versus 1.6% (95% CI:0.7-2.6) (*p *< 0.01)] respectively (Table 1).

**Table 1:** Characteristics for women who tested positive for *Chlamydia trachomatis *or *Mycoplasma genitalium*.

Variable	Participants' characteristics N (%)	Chlamydia Prevalence (95%CI^b^) (no.^c ^positive/no. total women)	UOR^d ^(95% CI)	AOR^e ^(95% CI)	MG Prevalence (95% CI) (no. positive/no. total cases)	UOR (95% CI)	AOR (95% CI)
Age^a ^(median age)			0.9 (0.8, 1.0)	0.9 ( 0.8, 1.0)		1.0 (0.8, 1.3)	1.0 (0.8, 1.3)
Country of birth							
Not Australian born	121 (11.5)	1.7 (0.4, 6.7) (2/121)	1.0	1.0	1.7 (0.5, 5.2) (2/120)	1.0	1.0
Australian born	934 (88.5)	5.4 (3.5, 8.1) (50/934)	3.4 (0.7, 16.6)	3.1 (0.6, 14.7)	2.6 (1.5, 3.7) (24/929)	1.6 (0.4, 6.2)	1.4 (0.3, 5.9)
Indigenous status							
Not indigenous	1059 (97.7)	4.8 (3.2, 7.3) (51/1059)	1.0	1.0	2.4 (1.4, 3.4) (25/1059)	1.0	1.0
Indigenous	25 (2.3)	4.0 (0.5, 27.2) (1/25)	0.8 (0.1, 6.0)	1.0 (0.1,8.0)	8.0 (2.4, 23.2) (2/25)	3.6 (0.9, 13.5)	4.5 (1.4, 14.9)
Education							
up to year 12^f^	609 (56.1)	5.9 (2.9, 8.9) (36/609)	1.0	1.0	3.1 (1.8, 4.4) (19/604)	1.0	1.0
Tertiary	477 (43.9)	3.6 (1.4, 5.7) (17/477)	0.6 (0.3, 1.2)	0.6 (0.3, 1.3)	1.7 (0.4, 2.9) (8/476)	0.5 (0.2, 1.1)	0.6 (0.3, 1.2)
Employment							
Unemployed/Not working	418 (38.5)	4.8 (1.8, 7.8) (20/418)	1.0	1.0	2.4 (0.7, 4.1) (10/416)	1.0	1.0
Employed	668 (61.5)	4.9 (2.7, 7.1) (33/668)	1.0 (0.6, 1.9)	1.0 (0.5, 1.9)	2.5 (1.6, 3.5) (17/664)	1.1 (0.5, 2.1)	1.0 (0.5, 2.1)
Clinic type							
GP	738 (66.1)	3.4 (1.5, 5.3) (25/738)	1.0	N/A^g^	1.6 (0.7, 2.6) (12/735)	1.0	N/A^g^
SHC/FP	378 (33.9)	7.9 (4.1, 11.8) (30/378)	2.5 (1.2, 4.9)		4.0 (2.7, 5.3) (15/375)	2.5 (1.4, 4.6)	
No. partners last 12 months							
<2	553 (51.8)	1.3 (0.5, 2.1) (7/553)	1.0	1.0	1.4 (0.6, 2.3) (8/551)	1.0	1.0
2+	515 (48.2)	8.5 (5.6, 11.5) (44/515)	7.3 (4.3, 12.2)	6.4 (3.6, 11.3)	3.7 (2.1, 5.3) (19/511)	2.6 (1.3, 5.3)	2.2 (1.0, 4.6)
Partners 12 months without condoms							
0	301 (29.0)	3.0 (1.5, 6.1) (9/301)	1.0	1.0	0.3 (0.0, 2.3) (1/299)	1.0	1.0
1-2	599 (57-8)	4.3 (2.6, 6.6) (26/599)	1.5 (0.8, 2.9)	1.4 (0.7, 2.6)	2.5 (1.6, 3.9) (15/595)	7.7 (1.0, 62.4)	7.2 (0.9, 57.6)
3+	137 (13.2)	10.9 (6.4, 18.0) (20/216)	4.0 (1.6, 10.1)	3.1 (1.0, 9.5)	6.6 (3.7, 11.5) (9/137)	20.9 (2.6, 167.3)	16.6 (2.0, 138.0)
Past history of Chlamydia diagnosis						1.0	1.0
No	965 (89.4)	4.2 (2.2, 6.3) (41/965)	1.0	1.0	2.4 (1.4, 3.4) (23/960)	1.5 (0.6, 4.0)	1.3 (0.4, 3.6)
Yes	114 (10.6)	9.6 (5.1, 14.2) (11/114)	2.4 (1.2, 4.7)	2.0 (1.1, 3.9)	3.5 (0.3, 6.8) (4/113)		
Antibiotics in last 2 months							
No	807 (74.0)	5.7 (3.0, 8.4) (48/833)	1.0	1.0	2.5 (1.6, 3.9) (21/802)	1.0	1.0
Yes	283 (26.0)	2.5 (0.5, 4.4) (7/283)	0.4 (0.2, 1.0)	0.4 (0.2, 1.0)	2.1 (0.8, 5.5) (6/282)	0.8 (0.3, 2.6)	0.8 (0.3, 2.5)

^a ^Represents change in odds of infection with each additional year of age. ^b ^CI: confidence interval. ^c ^no.= number. ^d ^Unadjusted odds ratio. ^e ^Adjusted Odd Ratio: Adjusted for clinic type the participants were recruited from. ^f ^Year 12 is the final year of secondary education in Australia. ^g ^Data not available.

Chlamydia was associated with younger age [AOR:0.9 (95% CI: 0.8, 1.0)] whereas MG was not. MG was associated with Indigenous status [AOR:4.5 (95% CI:1.4, 14.9)] (n = 2); a strong association was found between chlamydia infection and increased numbers of sexual partners. The odds of testing positive for chlamydia were six times greater for women who had had two or more sexual partners in the preceding year compared with women with fewer partners [AOR:6.4 (95% CI:3.6, 11.3)], the association was not as strong for MG [AOR:2.2 (95% CI:1.0, 4.6)]. In contrast, the odds of infection associated with the reported number of unprotected sex partners was far greater for MG [≥3 unprotected sex partners in the last 12 months: AOR:16.6 (95% CI:2.0, 138.0)] than for chlamydia [≥3 unprotected sex partners in the last 12 months: AOR:3.1 (95% CI:1.0, 9.5)]. Having being diagnosed with chlamydia in the past was also associated with testing positive for chlamydia [AOR:2.0 (95% CI:1.1, 3.9)], but not for MG (Table 1).

Self-reported use of any antibiotic in the prior two months was inversely associated with chlamydia [AOR:0.4 (95% CI:0.2, 1.0)], but was not associated with MG [AOR:0.8 (95% CI:0.3, 2.5)]. There were no associations with any other demographic characteristics collected (country of birth, employment status or education level) and chlamydia or MG (Table 1).

Chlamydia was not associated with any self-reported genital symptoms but MG was associated with self-reported 'abnormal vaginal discharge' [AOR:2.1 (95% CI:1.1, 4.0)] (Table 2). Women testing positive for MG reported a greater number of symptoms on average than women testing positive for chlamydia, although this did not reach statistical significance (1.9 symptoms versus 1.3; *p *= 0.2).

**Table 2:** Associations between self-reported symptoms and infection with *Chlamydia trachomatis *or *Mycoplasma genitalium *(MG).

Symptoms in last month	Chlamydia % (95% CI^a^) (n/no. women)	UOR^b^ (95% CI)	AOR^c^ (95% CI)	MG % (95% CI) (n/no. women)	UOR (95% CI)	AOR (95% CI)
Abnormal discharge						
No	4.6 (2.9, 7.2) (40/840)	1.0	1.0	1.8 (1.1, 2.8) (15/836)	1.0	1.0
Yes	4.8 (2.9, 7.8) (12/249)	1.0 (0.6, 1.7)	0.9 (0.6, 1.5)	4.0 (1.8, 6.2) (10/248)	2.3 (1.2, 4.6)	2.1 (1.1, 4.0)
Abnormal vaginal odour						
No	4.3 (2.8, 6.5) (40/902)	1.0	1.0	2.0 (1.3, 3.2) (18/898)	1.0	1.0
Yes	6.4 (3.0, 13.4) (12/187)	1.5 (0.7, 3.3)	1.4 (0.6, 3.1)	3.8 (1.7, 8.0) (7/186)	1.8 (0.7, 4.5)	1.7 (0.7, 4.2)
Burning when passing urine						
No	4.3 (2.7, 6.6) (39/888)	1.0	1.0	2.3 (1.6, 3.3) (20/883)	1.0	1.0
Yes	6.5 (3.6, 11.4) (13/201)	1.5 (0.9, 2.8)	1.5 (0.8, 2.6)	2.5 (1.2, 5.3) (5/201)	1.0 (0.5, 2.2)	1.0 (0.5, 2.1)
Abdominal pain						
No	4.6 (3.0, 6.9) (36/770)	1.0	1.0	2.0 (1.2, 3.1) (15/762)	1.0	1.0
Yes	5.0 (2.8, 8.8) (16/319)	1.1 (0.7, 1.8)	1.1 (0.6, 1.8)	3.1 (1.8, 5.4) (10/318)	1.6 (0.8, 3.4)	1.6 (0.7, 3.3)
Dyspareunia						
No	5.1 (3.2, 7.9) (45/849)	1.0	1.0	2.1 (1.4, 3.1) (18/860)	1.0	1.0
Yes	3.2 (1.5, 6.8) (7/220)	0.6 (0.3, 1.4)	0.6 (0.2, 1.4)	3.2 (1.6, 6.3) (7/220)	1.5 (0.7, 3.3)	1.5 (0.7, 3.1)
Intramenstrual bleeding						
No	4.8 (3.2, 7.3) (43/874)	1.0	1.0	2.0 (1.3, 3.0) (17/865)	1.0	1.0
Yes	4.2 (2.3, 7.5) (9/215)	0.9 (0.5, 1.4)	0.8 (0.5, 1.3)	3.7 (1.8, 7.4) (8/215)	1.9 (0.8, 4.6)	1.8 (0.7, 4.4)
Number of symptoms:						
0	4.1 (2.6, 6.4) (19/461)	1.0	1.0	1.3 (0.6, 2.6) (6/456)	1.0	1.0
1	5.0 (2.7, 9.0) (11/222)	1.2 (0.7, 2.2)	1.1 (0.6, 2.1)	2.7 (1.4, 5.2) (6/222)	2.1 (0.8, 5.6)	2.0 (0.8, 5.2)
2	6.4 (3.2, 12.2) (13/204)	1.6 (0.8, 3.2)	1.5 (0.7, 3.0)	2.5 (0.1, 5.9) (5/204)	1.9 (0.5, 7.0)	1.8 (0.5, 6.4)
3	5.3 (2.4, 11.2) (5/95)	1.3 (0.5, 3.4)	1.2 (0.5, 3.1)	4.3xi (1.7, 9.9) (4/94)	3.3 (0.9, 12.1)	3.1 (0.8, 11.2)
4	4.6 (1.6, 12.8) (3/65)	1.1 (0.5, 2.8)	1.1 (0.4, 2.7)	1.5 (0.2, 10.8) (1/65)	1.2 (0.1, 10.9)	1.1 (0.1, 10.1)
5	3.3 (0.5, 20.8) (1/30)	0.8 (0.1, 6.4)	0.7 (0.1, 4.9)	10.0 (3.3, 26.5) (3/30)	8.3 (2.1, 32.5)	7.0 (1.9, 25.5)
6	0.0 (0/11)			0.0 (0/11)		

^a ^CI: confidence interval. ^b^Unadjusted odds ratio. ^c^Adjusted Odd Ratio: Adjusted for clinic type the participants were recruited from.

### Infectious load and serovars

**Figure 1:**
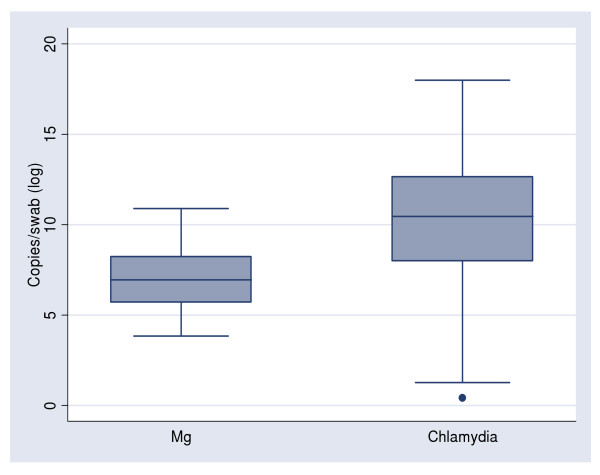
***Chlamydia trachomatis *(chlamydia) organism load and *Mycoplasma genitalium *(MG) organism load per swab (log)**.

Overall, 52 chlamydia-positive samples and 22 MG positive samples were analysed to determine their respective infectious loads. The median MG organism load was 100 times lower (5.7 × 10^4^/swab) than the median chlamydia organism load (5.6 × 10^6^/swab) (*p *< 0.01), and the quantitative range reported for MG was smaller (1.9 × 10^3^/swab to 2.1 × 10^6^/swab) than for chlamydia (4.2 × 10^3^/swab to 2.6 × 10^9^/swab) (Figure 1) There were no associations between self-reported symptoms, a past history of chlamydia or age and organism load for either chlamydia or MG (data not shown).

**Figure 2:**
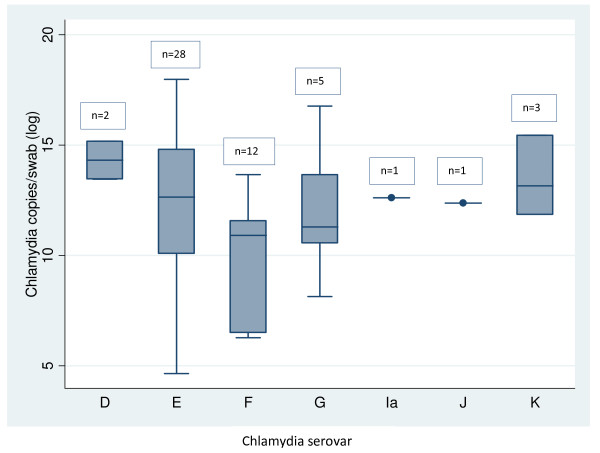
***Chlamydia trachomatis *(chlamydia) organism load per swab (log) for each chlamydia serovar detected**.

The chlamydia serovar was identified for 52 of 55 positive chlamydia specimens and 27 (51.9%) of these were serovar E and shared a 100% homology in their *omp1 *gene sequencing (Table 3). No cases of mixed chlamydia serovar were detected. The organism load was significantly higher for serovar D than serovar E (*p *= 0.04) and higher for serovar E than for serovar F (*p *= 0.06). No other differences were found (Figure 2) and no associations between chlamydia serovar and age were found (data not shown).

**Table 3:** Serovars detected in *Chlamydia trachomatis *positive samples.

		Nucleotide	Variant change			Genbank accession number	
**Chlamydia genotype**	**Frequency N (%)**	**Position^a^**	**Nucleotide**	**Amino acid**	**Reference strain**	**Reference**	**This study**

D	2 (3.6)	-	-	-	D-IC-CAL8	DQ06428	HM230054
E	27 (49.0)	-	-	-	E-Bour	X52557	HM230055
E variant^b^	1 (1.8)	372	C > T	Silent (C)	E-Bour	X52557	HM230056
F	12 (21.8)	-	-	-	F-IC-Cal13	X52080	HM230057
G	3 (5.5)	-	-	-	G-DK-K1	AM90115	HM230058
G variant^c^	2 (3.6)	487; 1003	A > G; T > G	S > G; S > A	G-DK-K1	AM90115	HM230059
Ia	1 (1.8)	-	-	-	Ia-IU-TC0167ut	FJ26194	HM230061
J	1 (1.8)	-	-	-	J-UW-36	DQ06429	HM230062
K	3 (5.5)	-	-	-	K-UW-31	DQ06429	HM230063
N/A^d^	2 (3.6)						

**Total**	**55**						

a - based on reference strain *omp1 *sequence

b - E variant has 100% homology to Genbank sequence GU903922 (*C. trachomatis *strain 1969 from Australian MSM population)

c - G variant has 100% homology to Genbank sequence FJ261928 (G/IU-FW0267)

d - N/A: serovar unable to be determined.

### MG test-of-cure

Of the 27 women who were sent a second swab in the mail for an MG test-of-cure one month after azithromycin treatment, 20 (74%) returned swabs for testing. Of these, 17 tested negative, and three (15%) tested positive. The three women with persistent MG had further telephone consultations with a sexual health clinician who determined all had adhered to treatment, two had had partners concurrently treated with 1 g azithromycin, and one had no sexual contact since her diagnosis. These women were considered likely to have had treatment failure rather than a new infection [azithromycin failure: 15% (95% CI:3.2, 37.9)] and were prescribed 400 mg moxifloxacin daily for ten days. A second test-of-cure was performed one month later on two of the three women and both were negative, but the third woman failed to return a second test-of-cure.

## Discussion

This paper presents the largest community-based estimate of chlamydia prevalence among young Australian women and Australia's first MG prevalence survey. Consistent with previous international reports, we found the chlamydia prevalence (4.9%) was higher than the MG prevalence (2.4%) among young women [9-11]. We also found some important clinical and epidemiological differences between chlamydia and MG in this cohort, suggesting different transmission dynamics between the two infections.

Firstly, it is possible that MG is less infectious than chlamydia requiring a greater "exposure" or direct genital or cervical contact to acquire MG. This is supported by the 100 fold lower organism load among samples from women with MG compared with chlamydia, and the finding that MG was more strongly associated with unprotected sex than chlamydia. Clearly, further partner studies are needed to investigate the transmission dynamics for MG and chlamydia to determine if and how transmission dynamics differ.

The clinical features associated with MG and chlamydia also differed, MG was associated with vaginal discharge, but chlamydia showed no associations with any reported symptoms. Studies of the association between MG and specific genital symptoms have been somewhat conflicting with some studies determining an association between MG and genito-urinary symptoms including vaginal discharge and dysuria [28], and other studies finding no association with symptoms [29]. Overall, published data suggests that MG appears to be somewhat similar to chlamydia [10,29-31]. Further to this, no associations were found between organism load and reported symptoms for either chlamydia or MG, which was also consistent with the other studies [30].

Younger women were more likely to have a prevalent chlamydia infection which is consistent with other research [2], although younger age was not associated with MG infection.

Antibiotic use in the two months prior to being tested demonstrated a protective effect against chlamydia but not for MG. This is most likely because chlamydia has been shown to be sensitive to a number of commonly prescribed antibiotics [32], and MG is less likely to be sensitive to the same prescribed antibiotics [23,32-34].

We also found that MG but not chlamydia was associated with Indigenous status (Australian Aboriginal and Torres Strait Islander women). The number of Indigenous women in our study (n = 25) limited further exploration in the analysis. Nonetheless, these are the first prevalence estimates for MG in Indigenous Australian women and given that STI rates are generally higher in Indigenous women in Australia this is not a surprising MG finding [35].

Unlike other studies, we did not find any associations between chlamydia organism load and age or past history of chlamydia infection [30,36]. We did find evidence to suggest that chlamydia serovar was associated with organism load. However, this was based on a small number of cases. Nevertheless, given that others have not found any association between serovar and organism load [30], and uncertainty remains as to whether serovar is associated with disease severity, further studies with larger sample sizes are needed to investigate serovar and organism load.

There were a number of limitations to our study. Firstly, our sample had a higher proportion of Australian-born, well-educated and sexually-active women than the general background population in Australia for the same age [26,27], however, these are common findings in similar research studies investigating sexual health issues [2,37]. It is difficult to assess the impact this may have had on our prevalence estimates because increased number of partners is often associated with increased prevalence [2,38] and higher education levels tend to be associated with reduced prevalence. We also were unsuccessful in recruiting 34% of the eligible women who were approached in the clinics, and while there were no associations between age and participation, we have no other information about the women we were unable to recruit. Nevertheless, this participation compares favorably with other chlamydia prevalence surveys [39-41].

Another limitation was relying on self-reported genital symptoms; these have been found to be highly subjective, non-specific and frequently poorly associated with cervical STIs. Self-reporting of genital symptoms on questionnaires also do not always correlate well with clinician elicited symptoms [42]. We audited the clinical notes of a sub-set of 100 women and found very poor correlation with genital symptoms reported in the clinical notes (data not shown). Women were far more likely to self-report symptoms on their study questionnaire than were recorded by their clinicians in their clinical notes at the time of recruitment.

There were limitations to the organism load analysis. Samples were self-collected and therefore the equal efficiency of sampling could not be assured, and as positive samples were subjected to a number of assays, the mean organism loads were not able to be normalized to number of cells per sample. However, we did find that the serovars detected in our study were consistent with those reported in international data (serovar E followed by serovar F) [30,43].

There were a number of strengths to this study including the large sample size, the high participation rate of 66% and the broad range of geographical locations and socio-economic areas from where the women were recruited. Also, considering 66% of the women were recruited from general practice, and between 80 to 90% of young Australian women visit a general practice clinic each year [4], the study method chosen was likely to provide a broadly representative sample.

The prevalence of chlamydia in our study was higher (4.9%) compared with the only other Australian population-based chlamydia prevalence study for women in the same age range (3.7%) [2] but was similar to a small community-based study [3], and other studies involving young women [44,45]. Importantly our data suggest chlamydia prevalence is still somewhat lower in Australia than some other countries, most notably the UK [46].

These are the first population data on MG prevalence in Australia and our findings are very similar to the population data to date from international studies[2.3% (95%CI:1.3, 3.2)] [10], and [3.4% (95% CI:2.7, 4.3)] [11], but are somewhat higher than a study in the U.S [0.8% (95% CI:0.4, 1.6)] [9]. As increasing evidence supports a role for MG in PID and tubal factor infertility, MG is emerging as an important treatable STI in women. Worryingly, consistent with other published studies, we found that 1 g of azithromycin appears to be 85% effective at best for uncomplicated MG [24,33]. Further, MG is less responsive to the doxycycline and cefoxitin based regimens used in the presumptive treatment with in women with PID [47]. Clearly our data provide evidence that MG is not uncommon in young women in Australia, and impetus is needed for the commercialization of a diagnostic assay to improve the management of MG. This study also contributes to our understanding of the MG organism load in clinical samples. However, further studies are needed to be done to understand this compared with other STIs such as chlamydia and if there is any relationship between copy number and pathogenicity.

## Conclusions

This is the first large and broadly representative chlamydia prevalence survey and first MG prevalence survey in Australian women. Chlamydia prevalence was high in young sexually-active women in Australia and largely asymptomatic, supporting the need for further chlamydia control activities. There is also a significant burden of MG in this population, but importantly, this study identified that there are important differences in the epidemiology of chlamydia and MG and possibly in the transmission dynamics of these two infections. This is important information which contributes to the scant population data on MG, an emerging pathogen in young women.

## List of abbreviations

STI: sexually transmissible infection; GP: General Practitioner; SHC: Sexual Health Centre; MG: *Mycoplasma genitalium*; PID: pelvic inflammatory disease.

## Competing interests

The authors declare that they have no competing interests.

## Authors' contributions

JW, managed and implemented the study, completed the analysis and led the writing; JH, was the principal investigator for the study, led analysis and conceived the study; CKF, BD, JKK, VK, FB, SG, JG, MYC, CSB, SG, KM, MP designed the study methodology; LG, was involved in the analysis; MYC, CSB, KM, were involved in the recruitment strategy and medical management of the participants, particularly in Victoria; SNT, JT, NT & SG managed, designed and implemented all microbial testing, serovar analysis and quantitation of *Chlamydia trachomatis *and *Mycoplasma genitalium*; BD, JKK, HB were involved in the recruiting of participants in New South Wales; MC and FB were involved in recruiting in the Australian Capital Territory; EU, & SW, contributed to the implementation and completion of the study; all authors were involved in writing and editing this article.

## Pre-publication history

The pre-publication history for this paper can be accessed here:

http://www.biomedcentral.com/1471-2334/11/35/prepub
